# Investigation of polymorphisms rs7903146 and rs12255372 in the TCF7L2 gene in biochemical markers of severity of type 2 diabetes mellitus (T2DM) in a sample of adults with T2DM

**DOI:** 10.1186/1753-6561-8-S4-P58

**Published:** 2014-10-01

**Authors:** Simone Morelo Dal Bosco, Cristiane dos santos Costa, Adriana Regina Bitello, Crislene Sippel, Rafaela Bastian, Maria Wollinger, Julio Dessoy, Olívia Bouchacourt, Rosangela Leipinitz, Veronica Contini, Claudete Rempel, Julia Genro

**Affiliations:** 1 Centro Universitário Univates, Lajeado, RS, Brazil

## Introduction

Diabetes mellitus type 2 (DM2) is a metabolic disease that involves complex multifactorial genetic and environmental factors [1]. Studies on the molecular genetics has suggested that many genes may be important in susceptibility to DM26. Recently, large-scale studies such as genome-wide associations studies (GWAS) have shown several important genes associated with T2DM. Among these genes, one of the most strongly associated with the disease is TCF7L2. This gene has many polymorphisms described, however the majority of studies indicate variants rs7903146 and rs12255372 as the most important within this gene.

## Methodological procedures

The sample consisted of 61 adults diagnosed with T2DM, from in the SIS-Hiperdia patient follow-up program. This is characterized as a continuing research entitled: "Identification of risk factors in diabetic and hypertensive users and nonusers of herbal *Bauhinia forficata *(paw-of-cow) registered in the SIS program Hiperdia/MS from 2009 to 2010, at the 16th Regional Health/RS". We analyzed some biochemical markers such as glucose, glycated hemoglobin (HbA1C) and C-reactive protein (CRP) using a Mindray BS120 analyzer. The variant rs7903146 (C/T) and rs12255372 (G/T) of the TCF7L2 gene were genotyped by Real Time PCR. A significance level of 5%.was used to declare associations. The study was approved by the Research Ethics committee at UNIVATES under number 110/11.

## Results and discussion

There were 61 subjects, of which 43 (70.5%) were female and 18 (29.5%) males. It was found that the T allele frequency was 0.34 and 0.33 respectively for the rs7903146 and rs12255372. The genotypic frequencies are in agreement with the expected Hardy- Weinberg equilibrium. According to Wilfred et al (2012) [2] the risk allele T (rs7903146) is strongly associated with the risk of T2DM, especially in Caucasians. Studies also show that carriers of the risk allele exhibit increased levels of some inflammatory markers such as CRP and Hb1Ac [3]. In our study, no significant associations were detected between the variants analyzed and the biochemical variables (glucose : rs7903146 p = 0.92, p = 0.96 rs12255372, Hb1Ac : rs7903146 p = 0.16, p = 0.09 and rs12255372 CRP: p = 0.50 rs7903146, rs12255372 p = 0.39)

## Conclusion

Studies indicate that the presence the T allele in these polymorphisms can influence in the risk of development and gravity of the T2DM. However, in this study, the allele T was not associated to outcomes biochemical in individuals with DM2. Failure to observe any significant result does not exclude the role of the gene as a modulator of disease severity.

**Table 1:** Association of TCF7L2 gene polymorphism and inflammatory markers in DM2

SNP *TCF7L2*	Genotype #	Glucose		Hb1AC		CRP	
		**Level**	**p value**	**Level**	**p value**	**Level**	**p value**

**rs 7903146 T>C**	**CC (25)**	**136.04**	**0.92**	**6.98**	**0.16**	**1.68**	**0.50**

	**CT (25)** **TT (07)**	**141.68** **139.85**		**6.3** **5.74**		**1.38** **0.67**	

**rs 12255372 T>G**	**GG (26)**	**137.03**	**0.95**	**7.02**	**0.09**	**1.58**	**0.39**

	**GT (28)** **TT (06)**	**140.71** **141.00**		**6.48** **5.36**		**1.25** **2.56**	
